# BTN3A3 inhibits the proliferation, migration and invasion of ovarian cancer cells by regulating ERK1/2 phosphorylation

**DOI:** 10.3389/fonc.2022.952425

**Published:** 2022-08-17

**Authors:** Sihan Chen, Zhangyun Li, Yanyan Wang, Shaohua Fan

**Affiliations:** ^1^ School of Life Science, Jiangsu Normal University, Xuzhou, China; ^2^ Department of Ultrasonic Medicine, The First People’s Hospital of Xuzhou, Xuzhou Municipal Hospital Affiliated to Xuzhou Medical University, Xuzhou, China

**Keywords:** BTN3A3, ovarian cancer, ERK1/2, FGF2, SCH772984

## Abstract

Butyrophilin Subfamily 3 Member A3 (BTN3A3) is a type I transmembrane protein belonging to the immunoglobulin (Ig) superfamily, which is expressed in many cancers. Clinical data show that ovarian cancer patients with high expression of *BTN3A3* have a longer survival time, but the mechanism of BTN3A3 in the occurrence and progression of ovarian cancer is still unclear. Here, we found that *BTN3A3* knockdown can promote the proliferation, migration and invasion of ovarian cancer cells, while overexpression of *BTN3A3* can inhibit the proliferation, migration and invasion of ovarian cancer cells. We analyzed the immunoprecipitated BTN3A3 complex by mass spectrometry and found that BTN3A3 binds to FGF2, and the overexpression of *BTN3A3* leads to a decrease in the protein level of FGF2, which in turn leads to a decrease in the level of phosphorylation of ERK1/2. By increasing the protein level of FGF2, it was found that the level of ERK1/2 phosphorylation also increased. Finally, the cancer promotion phenomenon caused by *BTN3A3* knockdown can be improved by using ERK1/2 inhibitor SCH772984. To sum up, BTN3A3 interacts with FGF2, which inhibits FGF2/ERK1/2 axis and ultimately inhibits the proliferation, migration and invasion of ovarian cancer cells. Our results suggest that BTN3A3 may be a prognostic marker and a potential therapeutic target for ovarian cancer.

## Introduction

Ovarian cancer is a highly malignant type of tumor. At present, it has been one of the main causes of death of gynecological cancer patients (1, 2). Ovarian cancer is the second most frequent cause of gynecologic cancer deaths in women worldwide (3). The high fatality rate of this malignant tumor is largely due to the fact that nearly 75% of patients with ovarian cancer are diagnosed at the advanced stage of the tumor and are accompanied by extensive abdominal metastasis (4). It has been reported that the incidence of ovarian cancer is related to fertility in women (5, 6), sex hormone stimulation (7), persistent ovulation (7), family genetic history (8–10), oral use of contraceptive (11, 12), age of first birth and menopause (13), but they are all uncertain factors. Currently, there are no very effective biomarkers for ovarian cancer (14, 15). Therefore, for ovarian cancer, a heterogeneous disease with complex molecular mechanisms and genetic changes (16), it is particularly important to study the molecular mechanism of its occurrence and progression.

Butyrophilin (BTN) family is a type I transmembrane protein belonging to the immunoglobulin (Ig) superfamily (17, 18). It has structural homology with B7 family members at the extracellular domain level and is considered to be B7 family related proteins (19). The BTN3A is a subfamily of BTN family, including BTN3A1, BTN3A2 and BTN3A3. At present, it has been confirmed that BTN3A1 can kill tumor cells by activating Vγ9Vδ2 T cells, which indicates that it is related to tumor immunity (20, 21), but little is known about BTN3A2 and BTN3A3. As far as BTN3A3 is concerned, studies have shown that it may play an important role in some types of cancer. For example, studies on gastric cancer have found that the expression of *BTN3A3* can help predict the sensitivity of gastric cancer patients to the chemotherapeutic drug fluorouracil (22), while in breast cancer, BTN3A3 can enhance the stemness of breast cancer cells through interaction with LSECtin (23). Among the related studies of ovarian cancer, some results show that the single nucleotide polymorphism (SNP) of *BTN3A3* is negatively correlated with the risk of ovarian cancer (24). However, the mechanism of BTN3A3 in the occurrence and progression of ovarian cancer is not known.

Fibroblast Growth Factor 2 (FGF2) is a member of the heparin-binding growth factor family. It is an important angiogenic molecule involved in tumor progression (25–27). Studies have shown that FGF2 is one of several growth factors that play a core role in ovarian carcinogenesis (25, 28). Compared with normal ovarian tissues, the expression of FGF2 in ovarian cancer tissues was significantly increased (29, 30), and negatively correlated with the overall survival time of patients with ovarian cancer (29). Some studies have shown that FGF2 is closely related to extracellular signal-regulated kinase 1/2 (ERK1/2)/MAPK pathway, which can promote the proliferation, migration and invasion of tumor cells through the activation of ERK1/2 (26, 30, 31). The results of studies in patients with ovarian cancer show that FGF2 is one of several growth factors that play a core role in the carcinogenesis of ovarian cancer (25), and it is also the main angiogenic factor expressed in ovarian cancer at mRNA level (28). After transcription, the mRNA of *FGF2* is translated into its polypeptide product-a complex set of four co-expressed isomers with apparent molecular weights of 24, 23, 22 and 18 kDa, respectively (32).

Clinical data show that ovarian cancer patients with high expression of BTN3A3 have a longer survival time, suggesting that BTN3A3 may play an important role in the occurrence and progression of ovarian cancer. Therefore, we knocked down or overexpressed *BTN3A3* in ovarian cancer cell lines to detect cell proliferation, migration and invasion ability, and used immunoprecipitation combined with mass spectrometry to explore the possible mechanism of BTN3A3 in the progression of ovarian cancer.

## Materials and methods

### Cell line

Human ovarian cancer cells ES-2 and NIH : OVCAR-3 were obtained from American Type Culture Collection (ATCC). HO-8910 and HO-8910PM cells were kindly provided by Cell Bank/Stem Cell Bank, Chinese Academy of Sciences (Shanghai, China). SK-OV-3 cells was obtained from Shanghai Zhong Qiao Xin Zhou Biotechnology Co., Ltd. (Shanghai, China).

### Lentivirus production

The *BTN3A3* short hairpin RNA (shRNA) lentivirus was produced by Cyagen Biosciences Inc. (Guangzhou, China). Oligonucleotides were synthesized to generate an annealing shRNA targeting the sequence of BTN3A3 from position 2238 to 2258 (5’-CCCTGTCGGGTAGTCAT ATTT-3’), from 1213 to 1233 (5’-GAGAAGTCTTTGGCCTATCAT-3’) and from 1254 to 1274 (5’-CAAACCTGCGGATGTGATTCT-3’).

The pWPXL-Puro-BTN3A3 or pWPXL-Puro plasmids was co-transfected into 293T cells with packaging plasmid psPAX2 and envelope plasmid pMD2.G, respectively. After 48 hours, the virus was collected and filtered to obtain *BTN3A3* overexpression lentivirus and control lentivirus. The plasmids and the specific oligonucleotide sequences used for plasmid construction are shown in **Table S1** and **Table S2**.

### Western blot analyses

Assays were performed as previously described (33). The antibodies used were listed in **Table S3**.

### Colony formation assay

The cells were dispersed into single cells and seeded into 60 mm culture dishes. After cultured in CO_2_ incubator for 2 weeks, the cells were stained with crystal violet staining and photographed.

### Cell Counting Kit-8 assay

The cells were seeded to a 96-well plate. On the 1st, 2nd, 3rd and 4th day, 10 μl CCK-8 reagent was added into each well and put back into the CO_2_ incubator for 1 hour. The absorbance at 450 nm was determined by Synergy H4 Hybrid Microplate Reader (BioTek, Winooski, VT, USA).

### Migration and invasion assay

The cells were suspended in serum-free medium and seeded in the upper layer of Transwell chamber, and the complete medium was added in the lower layer. In the invasion experiment, the Matrigel basement membrane matrix (BD Biosciences, MA, USA) should be laid in advance. After 24 hours, the cells passing through the membrane were stained with crystal violet, and then observed and photographed.

### Wound-healing assay

The cells were cultured in a six-well plate and scratched with the pipet tip when they grew to 95% fusion. After the cells were photographed, they were placed back into the CO_2_ incubator for 24 hours and photographed again.

### Co-immunoprecipitation assay

An appropriate amount of protease inhibitor (Roche Diagnostics GmbH, Mannheim, Germany) and phosphatase inhibitor (Beyotime Biotechnology, Shanghai, China) were added to Pierce IP Lysis Buffer (Thermo Scientific, Rockford, IL, USA). The above lysate was mixed and added to the cell culture dish, scraped off the cells with the cell scraper, and lysed the cells on the ice. Protein A/G PLUS-Agarose beads (sc-2003, Santa Cruz Biotechnology, Dallas, TX, USA) was added to the lysate and shaken on a rotating shaker at 4°C for 1 hour to remove non-specific proteins. After centrifugation at 4°C for 5 minutes, Protein A/G PLUS-Agarose beads was removed. The primary antibody or control IgG (**Table S3**) was added to the lysate, shaken on a rotating shaker at 4°C for 2 hours, and incubated overnight with Protein A/G PLUS-Agarose beads. The beads were washed three times at 4°C with IP Lysis Buffer for 5 minutes each time. Then 2 × loading buffer was added and boiled at 95°C for 10 minutes to separate the protein from beads. The supernatant was collected for Western blot analyses.

### Mass spectrometry

As mentioned above, the co-immunoprecipitation experiment was carried out with Flag-tag antibody, and the final collected supernatant was used for sodium dodecyl sulfate-polyacrylamide gel electrophoresis (SDS-PAGE), and the gel was stained with Coomassie Brilliant Blue staining solution. The gel with the sample was cut off and used for mass spectrometric analysis (Gene Denovo, Guangzhou, China).

### Immunofluorescence

The cells on the cover glass were fixed with 4% paraformaldehyde and permeabilized by 0.5% Trixton-100, then blocked with 5% BSA for 30 minutes. The cells were incubated with primary antibody (**Table S3**) at 37°C for 1 hour. After washing with PBST, the cells were incubated with the Alexa Fluor-conjugated secondary antibody (**Table S3**) at room temperature for 2 hours, and then incubated with 4’, 6-diamidino-2-phenylindole (DAPI) for 3 minutes to detect the nuclei. The cells were observed and photographed by confocal microscope (Leica TCS SP8, Leica Microsystems, Solms, Germany).

### Statistical analysis

Data are presented as mean ± SEM and comparisons were made using one-way analysis of variance (ANOVA) or Student’s *t* test. All *P*-values below 0.05 were considered significant.

## Results

### Expression level of BTN3A3 in ovarian cancer cell lines and establishment of stable transfection cell lines

**Figure 1:**
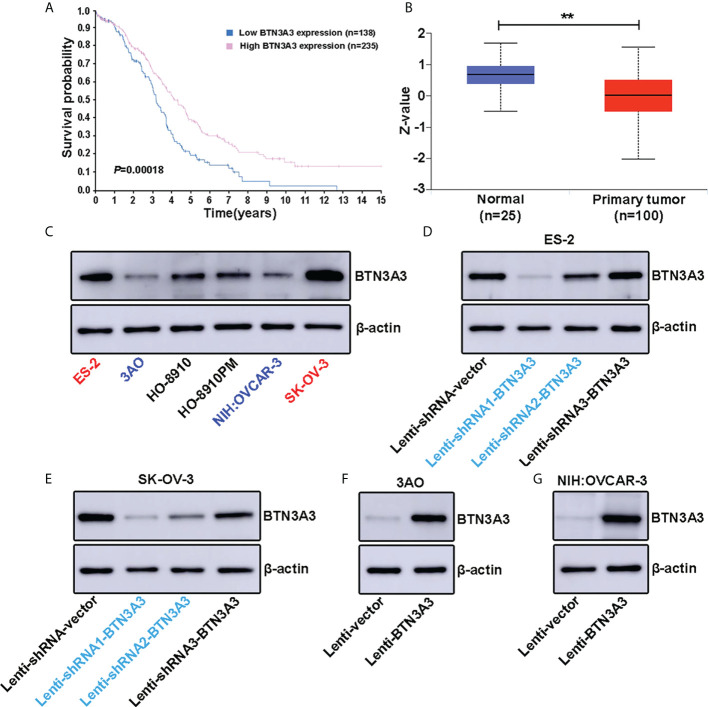
Expression level of BTN3A3 in ovarian cancer cell lines and establishment of stable transfection cell lines. **(A)** The Human Protein Atlas (HPA) website (https://www.proteinatlas.org/ENSG00000111801-BTN3A3/pathology/ovarian+cancer) shows the relationship between *BTN3A3* expression and the prognosis of patients with ovarian cancer. **(B)** The CPTAC database analysis results on the UALCAN website (https://ualcan.path.uab.edu/cgi-bin/CPTAC-Result.pl?genenam=BTN3A3&ctype=OV) show that the protein level of BTN3A3 differs between normal ovarian and ovarian cancer tissues. The expression of BTN3A3 in six ovarian cancer cell lines was detected by Western blot **(C)**. Western blot was also used to detect the effect of BTN3A3 knockdown in ES-2 **(D)** and SK-OV-3 **(E)** cells and the overexpression of BTN3A3 in 3AO **(F)** and NIH : OVCAR-3 **(G)** cells. ** *P* < 0.01.

Human Protein Atlas (HPA) provides researchers with biomedical resources based on clinical pathology, from which we found that ovarian cancer patients with high expression of *BTN3A3* had a longer survival (*P* = 0.00018) (**Figure 1A**), suggesting that BTN3A3 may play an important role in the occurrence and progression of ovarian cancer. In addition, the analysis of Clinical Proteomic Tumor Analysis Consortium (CPTAC) clinical database on UALCAN website showed that the protein level of BTN3A3 in ovarian cancer was significantly lower than that in normal ovarian tissue (*P* = 0.0013) (**Figure 1B**), including 25 normal ovarian samples and 100 ovarian cancer samples. In order to understand the expression level of BTN3A3 in different ovarian cancer cells, Western blot (**Figure 1C**) were used to detect the protein level of BTN3A3 in six ovarian cancer cell lines (ES-2, 3AO, HO-8910, HO-8910PM, NIH : OVCAR-3 and SK-OV-3). Then, we selected two ovarian cancer cell lines ES-2 and SK-OV-3 with high expression of BTN3A3. The two cell lines were infected with lentivirus encoding shRNA targeting *BTN3A3*, and their knockdown efficiency was detected by Western blot (**Figures 1D, E**). The results showed that the interference effect of BTN3A3-shRNA1 and BTN3A3-shRNA2 was better than that of BTN3A3-shRNA3 in ES-2 and SK-OV-3 cells. So we chose BTN3A3-shRNA1 and BTN3A3-shRNA2 to carry on the follow-up experiments. Then *BTN3A3* was overexpressed in 3AO and NIH : OVCAR-3 cells with lentivirus. The effect of overexpression was detected by Western blot (**Figures 1F, G**).

### Knockdown of *BTN3A3* promotes the proliferation, migration and invasion of ovarian cancer cells

**Figure 2:**
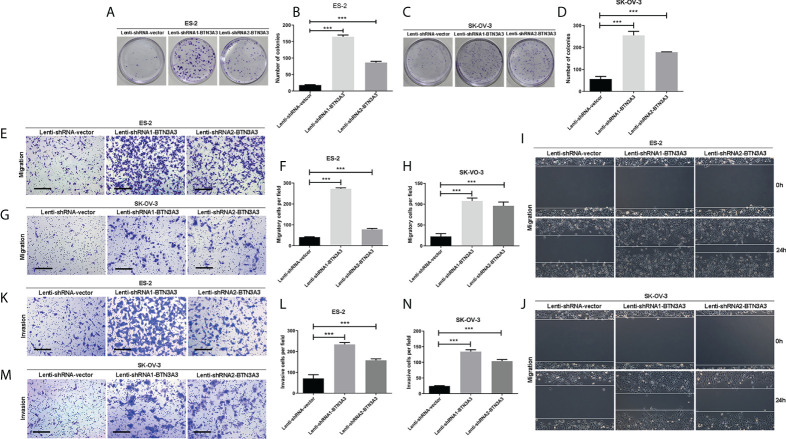
Knockdown of *BTN3A3* promotes the proliferation, migration and invasion of ovarian cancer cells. The colony formation assay was used to detect the proliferation ability of ES-2 **(A, B)** and SK-OV-3 **(C, D)** cells after *BTN3A3* knockdown. Transwell assay was used to detect the migration **(E-H)** and invasion **(K-N)** ability of ES-2 cells and SK-OV-3 cells. The migration ability of ES-2 **(I)** and SK-OV-3 **(J)** cells was also detected by wound-healing experiment. Data are expressed as mean ± SEM of three replicates. *** *P* < 0.001. **(E, G, K, M)** Scale bars: 50 µm.

We examined the effect of *BTN3A3* knockdown on cell proliferation by colony formation assay. The results showed that after *BTN3A3* knockdown, the proliferation ability of two cell lines ES-2 and SK-OV-3 cells was significantly higher than that of control cells (**Figures 2A–D**). Transwell migration assay (**Figures 2E-H**) and wound-healing assay (**Figures 2I, J**) were used to detect the migration ability of cells. The results showed that the migration ability of ES-2 and SK-OV-3 cells was significantly enhanced after *BTN3A3* knockdown. Transwell invasion assay showed that the invasion ability of ES-2 and SK-OV-3 was significantly enhanced after *BTN3A3* knockdown (**Figures 2K–N**).

### Overexpression of *BTN3A3* inhibits the proliferation, migration and invasion of ovarian cancer cells

**Figure 3:**
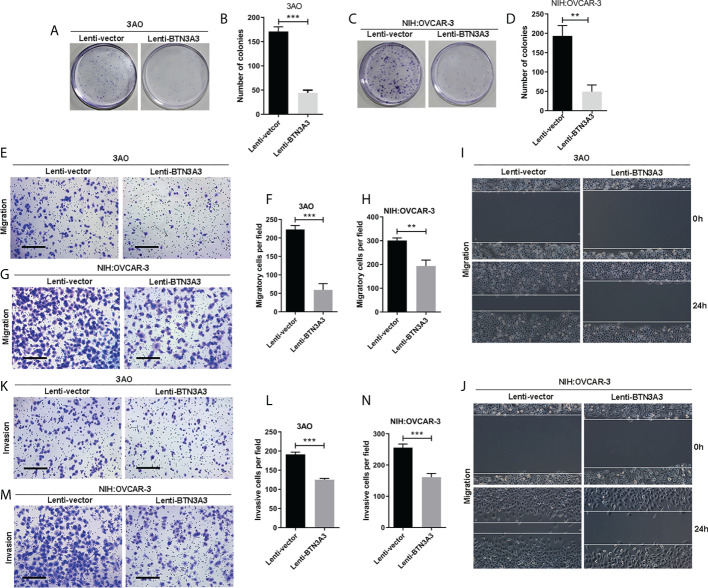
Overexpression of *BTN3A3* inhibits the proliferation, migration and invasion of ovarian cancer cells. The colony formation assay was used to detect the proliferation ability of 3AO **(A, B)** and NIH : OVCAR-3 **(C, D)** cells. Transwell assay was used to detect the migration **(E–H)** and invasion **(K-N)** ability of 3AO cells and NIH : OVCAR-3 cells. The migration ability of 3AO **(I)** and NIH : OVCAR-3 **(J)** cells was also detected by wound-healing experiment. Data are expressed as mean ± SEM of three replicates. **P < 0.01, ***P < 0.001. **(E, G, K, M)** Scale bars: 50 µm.

Similarly, we used clone formation assay (**Figures 3A–D**) to determine the changes in cell proliferation after overexpression of *BTN3A3*. In 3AO and NIH : OVCAR-3 cells, the proliferation ability of overexpressed *BTN3A3* cells was significantly inhibited compared with control cells. The results of Transwell migration assay (**Figures 3E–H**) and wound-healing assay (**Figures 3I, J** **)** showed that the migration ability of 3AO and NIH : OVCAR-3 cells decreased significantly after overexpression of *BTN3A3*. Transwell invasion assay showed that the invasion ability of 3AO and NIH : OVCAR-3 cells decreased significantly after overexpression of *BTN3A3* (**Figures 3K-N**).

### BTN3A3 binds to FGF2

**Figure 4:**
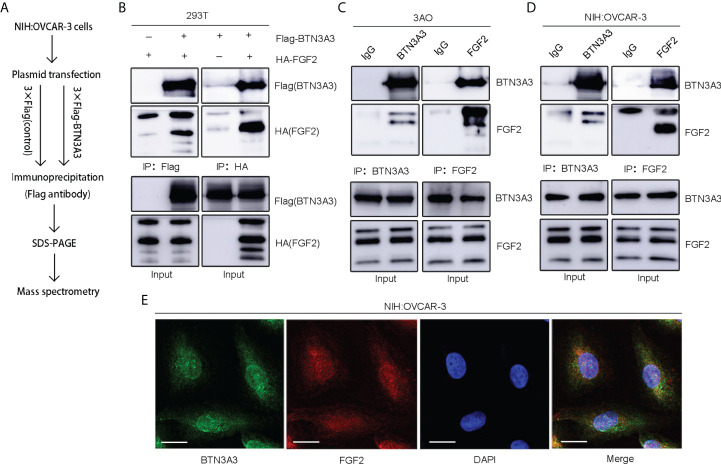
Identification of FGF2 as a binding protein of BTN3A3. **(A)** The Flag-labeled *BTN3A3* recombinant plasmid was transfected into NIH : OVCAR-3 cells. The cell lysate was co-immunoprecipitated with Flag-tag antibody, and the immunoprecipitation complex was detected by mass spectrometry. **(B)** 293T cells were transfected with Flag-BTN3A3 plasmid and HA-FGF2 plasmid respectively for co-immunoprecipitation to detect the exogenous binding of BTN3A3 and FGF2. The 3AO **(C)** and NIH : OVCAR-3 **(D)** cells were used for co-immunoprecipitation to detect the endogenous binding of BTN3A3 and FGF2. **(E)** Cellular immunofluorescence assay showed the co-localization of BTN3A3 and FGF2 in NIH : OVCAR-3 cells. Scale bars: 20 µm.

In order to further explore the mechanism of BTN3A3 inhibiting the progression of ovarian cancer, we constructed the *BTN3A3* overexpression plasmid with 3 × Flag tag and the control plasmid with 3 × Flag (**Table S1**), which were transfected into NIH : OVCAR-3 cells respectively. Then the co-immunoprecipitation assay was carried out, and the immunoprecipitation protein complex was analyzed by mass spectrometry (**Figure 4A**). Among the putative binding proteins (**Table S4**), we confirmed the interaction between BTN3A3 and FGF2 by plasmid co-transfection, followed by IP (immunoprecipitation) pull-down and IB (immunoblotting) detection. We co-transfected 3 × Flag-BTN3A3 and 3 × HA-FGF2 plasmids (**Table S1**) in 293T cells, and carried out exogenous co-immunoprecipitation assay (**Figure 4B**). Endogenous co-immunoprecipitation assay was carried out in 3AO and NIH : OVCAR-3 cells (**Figures 4C, D**). The results further confirmed the binding of BTN3A3 and FGF2. The results of immunofluorescence detection showed that BTN3A3 and FGF2 were co-located in NIH : OVCAR-3 cells (**Figure 4E**).

### FGF2 rescue increases phosphorylation of ERK1/2

**Figure 5:**
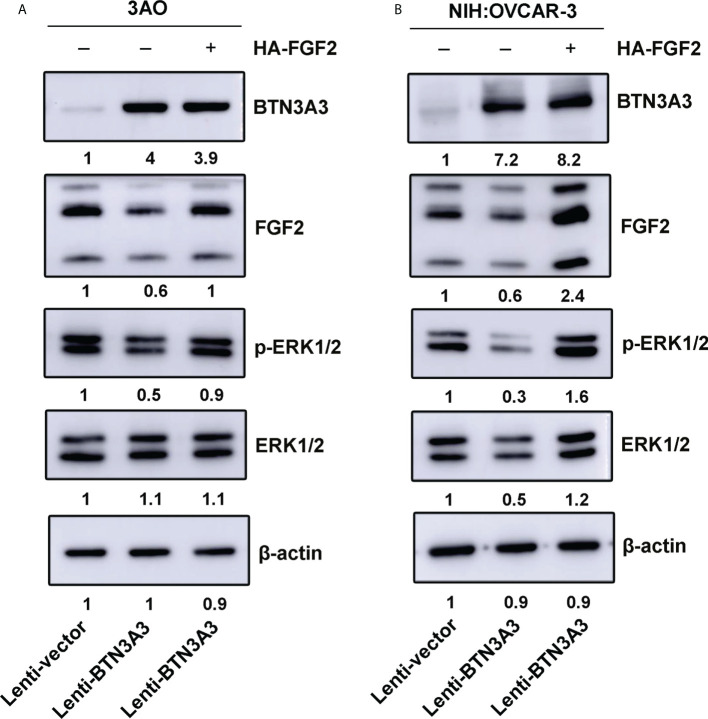
FGF2 rescue increases phosphorylation of ERK1/2. The *FGF2* recombinant plasmids were transfected into 3AO **(A)** and NIH : OVCAR-3 **(B)** cells respectively, and the changes of FGF2 protein level and ERK1/2 phosphate level were detected by Western blot.

After overexpression of *BTN3A3*, we found that the expression of *FGF2* decreased, and the phosphorylation of ERK1/2 also decreased. We used *FGF2* overexpression plasmid to rescue FGF2 in 3AO *BTN3A3* overexpression cells and NIH : OVCAR-3 *BTN3A3* overexpression cells respectively. The results showed that the level of ERK1/2 phosphorylation in *BTN3A3* overexpressed cells increased significantly after FGF2 rescue (**Figure 5**). Therefore, we found that the protein level of BTN3A3 could affect the activation of ERK1/2. In addition, we found that overexpression of *BTN3A3* in 3AO and NIH : OVCAR-3 cells significantly inhibited the proliferative capacity of the cells. In *BTN3A3* overexpressed cells, further increasing the level of FGF2 protein significantly increased the proliferative capacity of the cells (**Figure S1**).

### SCH772984 inhibits the proliferation, migration and invasion of *BTN3A3* knockdown cells

**Figure 6:**
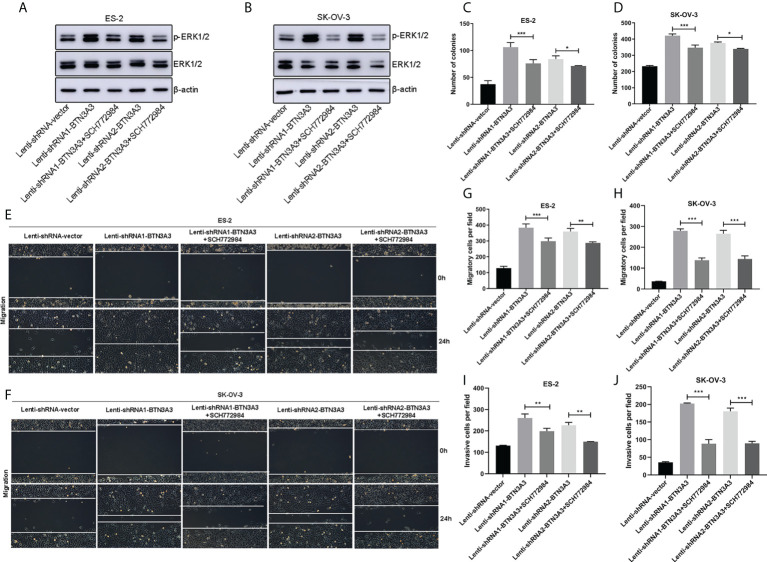
Effect of ERK1/2 inhibitor SCH772984 on ES-2 and SK-OV-3 cells after *BTN3A3* knockdown. In *BTN3A3* knockdown cells, ES-2 **(A)** and SK-OV-3 **(B)** were treated with SCH772984 with a final concentration of 100 nM, respectively, and the phosphorylation level of ERK1/2 was detected by Western blot. In *BTN3A3* knockdown cells, colony formation assay **(C, D)** showed the change of proliferation ability after treatment with SCH772984. Wound-healing assay **(E, F)** and Transwell migration assay **(G, H)** showed the change of migration ability after treatment with SCH772984 in *BTN3A3* knockdown cells, while the change of invasive ability was detected by Transwell invasion assay **(I, J)**. For the representative images of the results of colony formation assay and Transwell migration and invasion assays, please see **Figure S2**. Data are expressed as mean ± SEM of three replicates. *P < 0.05, **P < 0.01, ***P < 0.001.

We found that *BTN3A3* knockdown in ovarian cancer cells showed an increase in the level of ERK1/2 phosphorylation, so we treated the cells with ERK1/2 inhibitor SCH772984 (HY-50846, MedChemExpress, NJ, USA) at the final concentration of 100 nM, and found that the level of ERK1/2 phosphorylation decreased significantly (**Figures 6A, B**). The proliferation capability of SCH772984-treated BTN3A3-shRNA1 and BTN3A3-shRNA2 cells was examined using a colony formation assay. We found that the ability of cells to form colonies was significantly reduced (**Figures S2A, B**; **Figures 6C, D**). Then, we used the wound-healing experiment to further detect the migration ability of ovarian cancer cells. Compared with *BTN3A3* knockdown group, *BTN3A3* knockdown combined with SCH772984 treatment significantly inhibited the migration ability of ovarian cancer cells (**Figures 6E, F**). The Transwell migration assay showed the same results (**Figures S2C, D**; **Figures 6G, H**). Finally, we used Transwell invasion assay to detect the invasive ability of cells (**Figures S2E, F**; **Figures 6I, J**), results also showed that compared with *BTN3A3* knockdown group, *BTN3A3* knockdown combined with SCH772984 treatment significantly inhibited the invasive ability of cells.

## Discussion

The role of BTN3A3 in the occurrence and progression of ovarian cancer is unknown, but it is mentioned in only one article that *BTN3A3*’s SNP is negatively correlated with the risk of ovarian cancer (24). In the clinicopathological data of HPA, we found that *BTN3A3* expression was correlated with the prognosis of ovarian cancer. This makes us very interested in the relationship between BTN3A3 and ovarian cancer, so a series of studies have been carried out.

Firstly, we found that BTN3A3 can inhibit the proliferation, migration and invasion of ovarian cancer cells. Then we analyzed the immunoprecipitation BTN3A3 complex by mass spectrometry and found that FGF2 may bind to BTN3A3, which was further confirmed by a series of co-immunoprecipitation experiments. FGF2 is a relatively complex protein, and its mRNA is translated into a complex set of protein isomers with apparent molecular weights of 24, 23, 22 and 18 kDa, respectively (32). Most of our Western blot results show only three of these bands, while four bands are shown in 293T cells. It is speculated that 293T is a human embryonic kidney cell and expresses a wider range of proteins.

The combination of BTN3A3 and FGF2 suggests that BTN3A3 may play a role in regulating FGF2 signal. In addition, several laboratories have reported that there is a close relationship between the protein level of FGF2 and the phosphorylation level of ERK1/2 (19, 21, 30). The overexpression of *BTN3A3* leads to the decrease of FGF2 protein level. It is speculated that FGF2 may be degraded by ubiquitin or secreted outside the cell as a secretory protein, and the specific mechanism needs to be further studied. In order to further determine whether ERK1/2 is a downstream target of BTN3A3, we used ERK1/2 inhibitor SCH772984 to inhibit the increased phosphorylation of ERK1/2, caused by *BTN3A3* knockdown. Experiments on proliferation, migration and invasion have proved that inhibition of ERK1/2 activation can save cancer promotion caused by *BTN3A3* knockdown.

In addition, we also found that overexpression of *BTN3A3* could reduce the protein level of FGF2 in the nucleus of ovarian cancer cell line 3AO (**Figure S3**). It has been reported that the deletion of FGF2 in nucleus of pancreatic stellate cells significantly inhibits the invasive ability of pancreatic cancer cells (34). In human glioblastoma multiforme, *FGF2*’s nuclear import promoted the proliferation and survival of cancer cells (35). This proved that the decrease or increase of FGF2 in the nucleus may have an effect on the proliferation and invasion of cancer cells, but the cause of this phenomenon remains to be further studied.

To sum up, our studies have shown that BTN3A3 inhibits the proliferation, migration and invasion of ovarian cancer cells. BTN3A3 can bind to FGF2, regulate the protein level of FGF2, and then regulate the phosphorylation level of ERK1/2, thus affecting the proliferation, migration and invasion of ovarian cancer cells. These results suggest that BTN3A3 may play an important role in the progression of ovarian cancer, and may be a candidate protein to predict the prognosis of ovarian cancer patients and a potential therapeutic target for ovarian cancer.

## Data availability statement

The original contributions presented in the study are included in the article/**Supplementary Material**. Further inquiries can be directed to the corresponding authors.

## Author contributions

All authors were involved in the experiments or discussion for this paper. SC, ZL, and SF participated in the writing of the manuscript. All authors contributed to the article and approved the submitted version.

## Funding

This work was supported by the Xuzhou Municipal Science and Technology Project (KC20106 and KC21209); “333 Project” Award of Jiangsu Province (BRA2020252); “Six-one” Project for High-level Health Talents in Jiangsu Province (LGY2019057).

## Conflict of interest

The authors declare that the research was conducted in the absence of any commercial or financial relationships that could be construed as a potential conflict of interest.

## Publisher’s note

All claims expressed in this article are solely those of the authors and do not necessarily represent those of their affiliated organizations, or those of the publisher, the editors and the reviewers. Any product that may be evaluated in this article, or claim that may be made by its manufacturer, is not guaranteed or endorsed by the publisher.

## Supplementary material

Figure S1 FGF2 rescue promotes the proliferation of ovarian cancer cells. The *FGF2* recombinant plasmids were transfected into 3AO **(A)** and NIH : OVCAR-3 **(B)** cells respectively, and the change of proliferative ability was detected by CCK-8 assay. Data are expressed as mean ± SEM of four replicates. * represents the comparison between the Lenti-vector group and the Lenti-BTN3A3 group. # represents the comparison between the Lenti-BTN3A3 group and the Lenti-BTN3A3+FGF2 group. **P* < 0.05, ****P* < 0.001, ##*P* < 0.01, ###*P* < 0.001.

Figure S2 The representative images of the results of colony formation assay and Transwell migration and invasion assays. In *BTN3A3* knockdown cells, colony formation assay **(A, B)** showed the change of proliferation ability after treatment with SCH772984. Transwell migration assay **(C, D)** showed the change of migration ability after treatment with SCH772984 in *BTN3A3* knockdown cells, while the change of invasive ability was detected by Transwell invasion assay **(E, F)**. **(C–F)** Scale bars: 50 µm.

Figure S3 The overexpression of *BTN3A3* results in the decrease of FGF2 in the nucleus of 3AO cells. The results of cellular immunofluorescence assay showed that the FGF2 protein in the nucleus of 3AO cells decreased significantly after *BTN3A3* overexpression. Scale bars: 20 µm.
