# Supplementary figures and images for: BTN3A3 inhibits the proliferation, migration and invasion of ovarian cancer cells by regulating ERK1/2 phosphorylation
Source: Front Oncol. 2022 Aug 17;12:952425. doi: 10.3389/fonc.2022.952425 (PMC9428752; doi:10.3389/fonc.2022.952425)

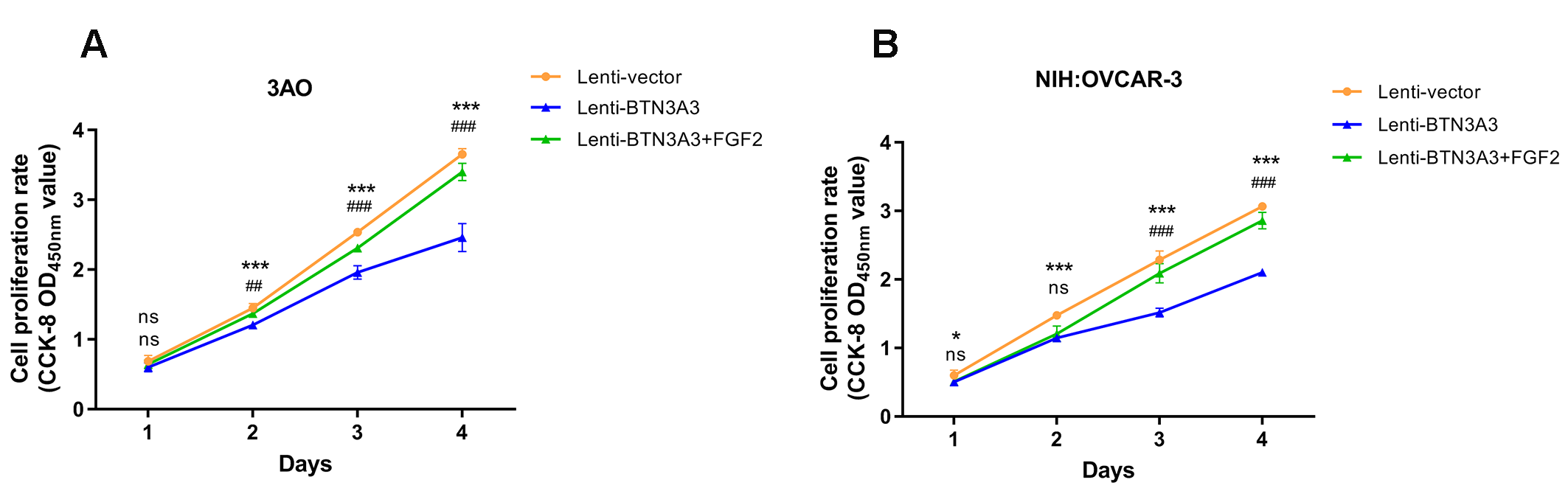

Supplement: Figure S1 — FGF2 rescue promotes the proliferation of ovarian cancer cells. The FGF2 recombinant plasmids were transfected into 3AO (A) and NIH : OVCAR-3 (B) cells respectively, and the change of proliferative ability was detected by CCK-8 assay. Data are expressed as mean ± SEM of four replicates. * represents the comparison between the Lenti-vector group and the Lenti-BTN3A3 group. # represents the comparison between the Lenti-BTN3A3 group and the Lenti-BTN3A3+FGF2 group. *P < 0.05, ***P < 0.001, ##P < 0.01, ###P < 0.001. [file Image_1.tif]

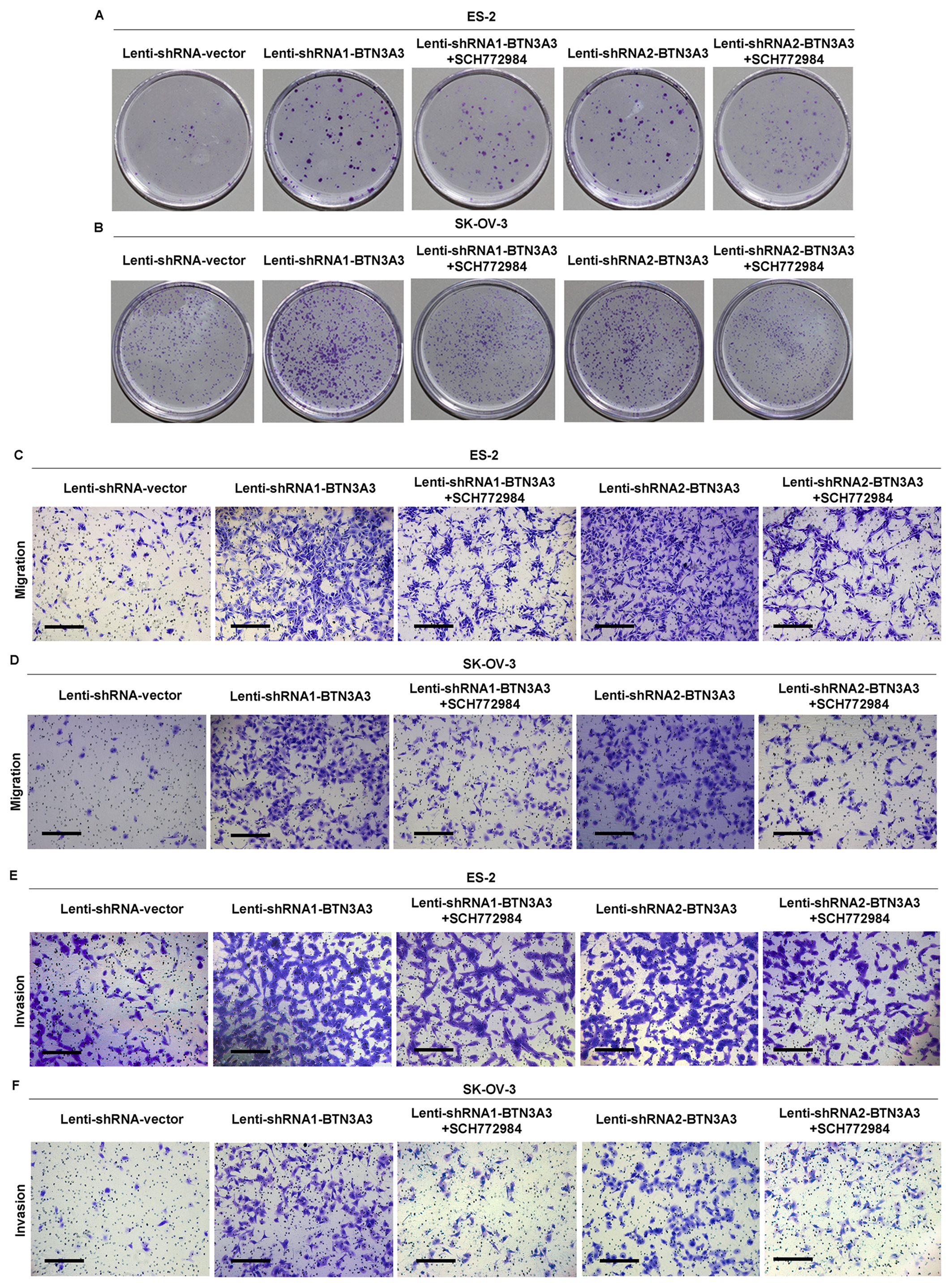

Supplement: Figure S2 — The representative images of the results of colony formation assay and Transwell migration and invasion assays. In BTN3A3 knockdown cells, colony formation assay (A, B) showed the change of proliferation ability after treatment with SCH772984. Transwell migration assay (C, D) showed the change of migration ability after treatment with SCH772984 in BTN3A3 knockdown cells, while the change of invasive ability was detected by Transwell invasion assay (E, F). (C–F) Scale bars: 50 µm. [file Image_2.tif]

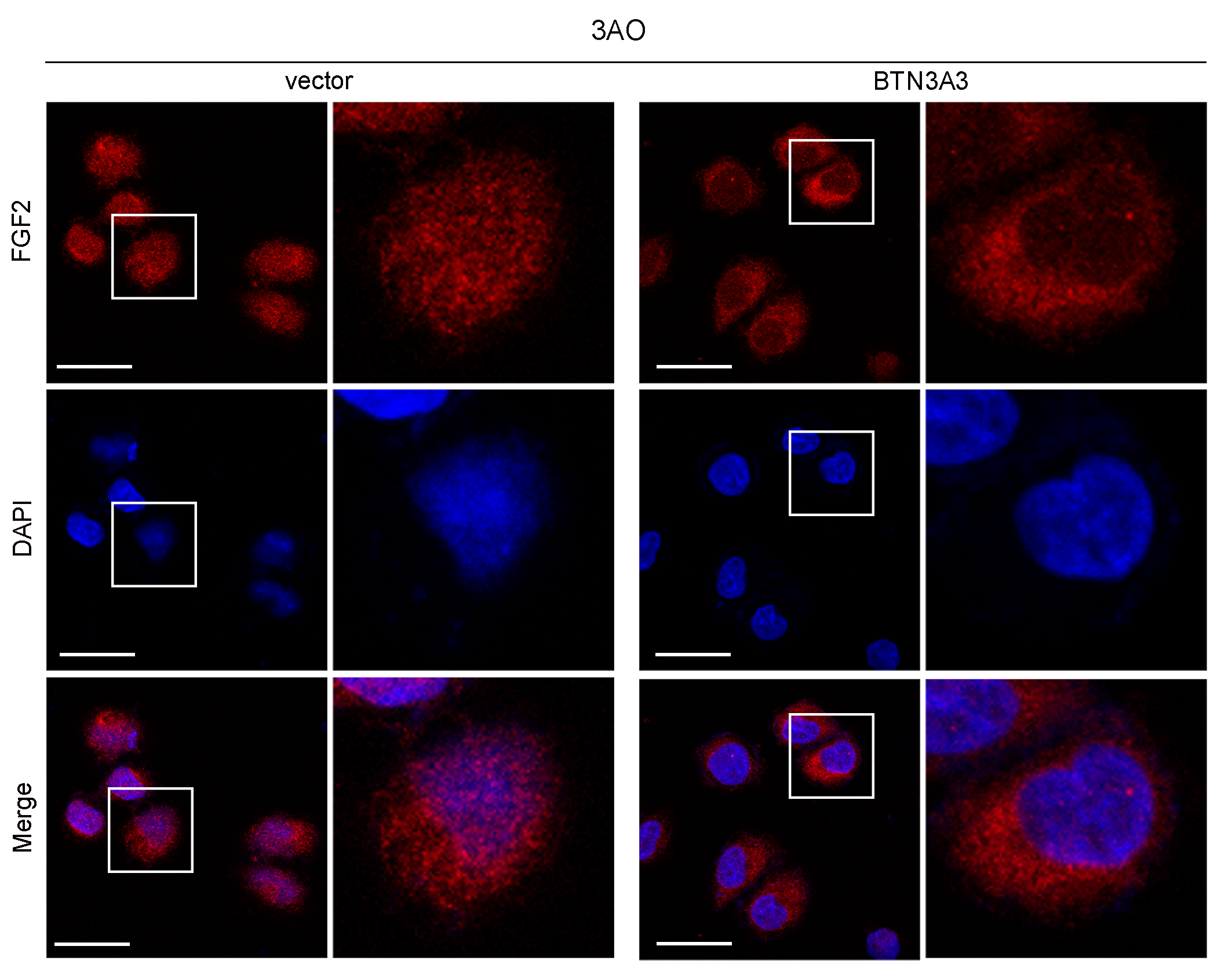

Supplement: Figure S3 — The overexpression of BTN3A3 results in the decrease of FGF2 in the nucleus of 3AO cells. The results of cellular immunofluorescence assay showed that the FGF2 protein in the nucleus of 3AO cells decreased significantly after BTN3A3 overexpression. Scale bars: 20 µm. [file Image_3.tif]
