# Supplementary material for: BTN3A3 inhibits the proliferation, migration and invasion of ovarian cancer cells by regulating ERK1/2 phosphorylation
Source: Front Oncol. 2022 Aug 17;12:952425. doi: 10.3389/fonc.2022.952425 (PMC9428752; doi:10.3389/fonc.2022.952425)
Supplement: Supplementary file 4 [file Table_1.docx]

**Table S1. The plasmids used for the construction and transfection**

| Plasmid | Description | Origin |
| --- | --- | --- |
| pWPXL | Plasmid construction | Addgene#12257, Cambridge, MA |
| pWPXL-Puro | Lentivirus production | Sangon, Shanghai, China |
| pWPXL-Puro-BTN3A3 | Lentivirus production | Sangon, Shanghai, China |
| psPAX2 | Lentivirus production | Addgene#12260, Cambridge, MA |
| pMD2.G | Lentivirus production | Addgene#12259, Cambridge, MA |
| pcDNA3.1-3×Flag | Immunoprecipitation | Sangon, Shanghai, China |
| pcDNA3.1-BTN3A3-3×Flag | Immunoprecipitation | Sangon, Shanghai, China |
| pcDNA3.1-FGF2 -3×HA | Immunoprecipitation | Sangon, Shanghai, China |
