# Supplementary material for: BTN3A3 inhibits the proliferation, migration and invasion of ovarian cancer cells by regulating ERK1/2 phosphorylation
Source: Front Oncol. 2022 Aug 17;12:952425. doi: 10.3389/fonc.2022.952425 (PMC9428752; doi:10.3389/fonc.2022.952425)
Supplement: Supplementary file 5 [file Table_2.docx]

| **Table S2. The sequences of the oligonucleotides for plasmid construct**   \| Name \| Sequence \| \| --- \| --- \| \| Lenti-shRNA-vector \| L: 5’- CCGGCCTAAGGTTAAGTCGCCCTCGCTCGAGCGA \| \|  \| GGGCGACTTAACCTTAGGTTTTTG -3’ \| \|  \| R: 5’- AATTCAAAAACCTAAGGTTAAGTCGCCCTCGCTC \| \|  \| GAGCGAGGGCGACTTAACCTTAGG -3’ \| \| Lenti-shRNA1-BTN3A3 \| L: 5’- CCGGCCCTGTCGGGTAGTCATATTTCTCGAGAAA \| \|  \| TATGACTACCCGACAGGGTTTTTG -3’ \| \|  \| R: 5’- AATTCAAAAACCCTGTCGGGTAGTCATATTTCTC \| \|  \| GAGAAATATGACTACCCGACAGGG -3’ \| \| Lenti-shRNA2-BTN3A3 \| L: 5’- CCGGGAGAAGTCTTTGGCCTATCATCTCGAGATG \| \|  \| ATAGGCCAAAGACTTCTCTTTTTG -3’ \| \|  \| R: 5’- AATTCAAAAAGAGAAGTCTTTGGCCTATCATCTCG \| \|  \| AGATGATAGGCCAAAGACTTCTC -3’ \| \| Lenti-shRNA3-BTN3A3 \| L: 5’- CCGGCAAACCTGCGGATGTGATTCTCTCGAGAGA \| \|  \| ATCACATCCGCAGGTTTGTTTTTG -3’ \| \|  \| R: 5’- AATTCAAAAACAAACCTGCGGATGTGATTCTCTC \| \|  \| GAGAGAATCACATCCGCAGGTTTG -3’ \| |
| --- | --- | --- | --- | --- | --- | --- | --- | --- | --- | --- | --- | --- | --- | --- | --- | --- | --- | --- | --- | --- | --- | --- | --- | --- | --- | --- | --- | --- | --- | --- | --- | --- | --- | --- |
