# Supplementary material for: BTN3A3 inhibits the proliferation, migration and invasion of ovarian cancer cells by regulating ERK1/2 phosphorylation
Source: Front Oncol. 2022 Aug 17;12:952425. doi: 10.3389/fonc.2022.952425 (PMC9428752; doi:10.3389/fonc.2022.952425)
Supplement: Supplementary file 6 [file Table_3.docx]

**Table S3. The proteins detected and the characteristics of the corresponding antibodies**

| Protein | Assay | | Research Resource Identifier (RRID) | Dilution |
| --- | --- | --- | --- | --- |
| BTN3A3 | | WB | Sigma-Aldrich, Cat# HPA007904, RRID:AB_1078307 | 1:2000 |
| BTN3A3 | | IHC | Sigma-Aldrich, Cat# HPA007904, RRID:AB_1078307 | 1:200 |
| BTN3A3 | | IF | Sigma-Aldrich, Cat# HPA007904, RRID:AB_1078307 | 1:200 |
| BTN3A3 | | IP | 15896-1-AP, Proteintech, IL, USA | 1:50 |
| FGF2 | | WB | ab208687, Abcam, Cambridge, UK | 1:1000 |
| FGF2 | | IF | Santa Cruz Biotechnology, Cat# sc-74412, RRID:AB_1122854 | 1:50 |
| FGF2 | | IP | Santa Cruz Biotechnology, Cat# sc-74412, RRID:AB_1122854 | 1:10 |
| p44/42 MAPK (ERK1/2) | | WB | Cell Signaling Technology, Cat# 4695, RRID:AB_390779 | 1:1000 |
| Phospho-p44/42 MAPK (ERK1/2) (Thr202/Tyr204) | | WB | Cell Signaling Technology, Cat# 4370, RRID:AB_2315112 | 1:1000 |
| HA-Tag | | WB | Cell Signaling Technology, Cat# 3724, RRID:AB_1549585 | 1:1000 |
| HA-Tag | | IP | 66006-2-lg, Proteintech, IL, USA | 1:50 |
| Flag-Tag | | WB | Sigma-Aldrich, Cat# F7425, RRID:AB_439687 | 1:1000 |
| Flag-Tag | | IP | AP0007, Bioworld Technology, St Louis Park, MN | 1:50 |
| β-actin | | WB | AP0060, Bioworld Technology, St Louis Park, MN | 1:10000 |
| Normal Mouse IgG | | IP | Santa Cruz Biotechnology, Cat# sc-2025, RRID:AB_737182 | 1:10 |
| Rabbit IgG | | IP | B900610, Proteintech, IL, USA | 1:50 |
| Anti-Rabbit IgG | | WB | Cell Signaling Technology, Cat# 7074, RRID:AB_2099233 | 1:2000 |
| Anti-Rabbit IgG Heavy Chain | | WB | A25222, Abbkine Scientific Co., Ltd, Wuhan, China | 1:5000 |
| Donkey Anti-Rabbit lgG H&L | | IF | Ab150073, Abcam, Cambridge, UK | 1:200 |
| Goat Anti-Mouse IgG H&L | | IF | Ab150120, Abcam, Cambridge, UK | 1:200 |
